# Rapid Web-Based Recruitment of Patients With Psoriasis: Multinational Cohort Study

**DOI:** 10.2196/44405

**Published:** 2023-06-20

**Authors:** Zacharias Duus Holm, John Robert Zibert, Simon Francis Thomsen, Ari Pall Isberg, Anders Daniel Andersen, Zarqa Ali

**Affiliations:** 1 Studies&Me A/S Copenhagen Denmark; 2 Department of Dermato-Venereology and Wound Healing Centre Copenhagen University Hospital Bispebjerg Copenhagen Denmark; 3 Future-Brain Aps Herlev Denmark

**Keywords:** web-based recruitment, remote recruitment, conversion rate, recruitment rate, psoriasis, population, dermatologist, derma, itchy skin, Insomnia Severity Index, ISI, Pittsburgh Sleep Quality Index, PSQI, Dermatology Life Quality Index, DLQI, quantile-quantile plot, Q-Q plot, dermatology

## Abstract

**Background:**

Wide-ranging patient recruitment not restricted to the location of the investigator will provide a better representation of the patient population in clinical studies.

**Objective:**

Our goal was to assess the feasibility of a broad web-based recruitment strategy in an 8-week observational study of 500 study participants with psoriasis and healthy controls from locations remote from the investigator and to assess the cost associated with each participant.

**Methods:**

A decentralized team in Denmark recruited patients with psoriasis and healthy controls using Google and Facebook advertisements and posts to Facebook groups. All individuals were screened via the internet, and patients diagnosed with psoriasis were included. Questionnaires regarding itch and sleep were completed by both groups at inclusion, week 4, and week 8.

**Results:**

During a 2-week recruitment period, 12,887 unique advertisement views were registered, and 839 participants were enrolled, of which 507 completed the study (220 with psoriasis and 287 healthy controls) with a retention rate of 60.4%. Participants were recruited from 11 different countries on 4 separate continents, mainly from the United States, Canada, and the United Kingdom. The recruitment rate was 59.9 participants per day, and the conversion rate was 57.2%. Recruitment costs were US $13 per enrolled participant and US $22 per participant completing the study.

**Conclusions:**

It is feasible and rapid to recruit a large number of participants from locations different from the investigator and to retain patients in an observational study with no visits to a clinical site at low costs.

## Introduction

Recruitment of study participants is considered to be one of the most difficult aspects of the clinical research process [1]. More than 3 out of 4 patients do not enroll in a given trial due to structural and clinical barriers [2]. Moreover, a disproportionate number of participants from higher socioeconomic classes are typically enrolled [3]. Globally, more than 80% of trials fail to meet the timeline for enrolling participants leading to the addition of study sites or extension of the respective study [3,4], and 30% of phase-3 trials are terminated due to enrollment difficulties [5].

In particular, recruitment of an insufficient number of participants in studies with rare diseases is a challenge. Internet advertising, especially through Facebook, has resulted in efficient enrollment of large numbers of individuals with rare diseases, compared to recruitment through government and academic websites, patient advocacy groups, and health care providers [6]. This indicates that internet communication advances provide new opportunities to assemble individuals with rare diseases to web-based patient registries from wide geographic areas for research. A larger and broader recruitment not restricted by geography will likely also provide a better representation of the patient population. In a review from 2020 [7], Facebook was the most used social media platform for recruiting study participants through web-based platforms for clinical trials.

Other benefits of web-based recruitment include lower cost and bigger outreach. Web-based recruitment costs are lower compared with in-person recruitment methods [8-10], and web-based recruitment may enable outreach to populations otherwise challenging to enroll [9-11].

The aim of this observational study was to assess the feasibility of a broad web-based recruitment not restricted by the location of the investigator. We aimed to recruit 500 study participants, including patients with psoriasis and healthy controls, and to assess the cost associated with each participant recruited via the internet.

## Methods

### Study Design and Participants

A team based in Copenhagen, Denmark, recruited patients with psoriasis and healthy controls located primarily in the United States, Canada, and the United Kingdom to an 8-week observational feasibility study using Facebook ads, Google Search AdWords, and posts to Facebook groups (Multimedia Appendix 1). Inclusion criteria were age >18 years and self-reported physician’s diagnosis of psoriasis. Healthy controls were individuals without a psoriasis diagnosis. Participants did not receive any compensation for participation.

### Data Collection

After signing up with contact information, participants received a screening and demographic questionnaire, including questions regarding age, gender, nationality, English language proficiency, comorbidities, if they suffered from itchy skin, medications (which medication they take and how often they take them), height, and weight. Patients with psoriasis were asked to estimate the percentage of body area affected by psoriasis, the number of years they have been living with psoriasis, self-rated severity of psoriasis (ie, mild, moderate, or severe), and the type of psoriasis they have (the full questionnaire could be viewed in Multimedia Appendix 2).

Validated questionnaires were adapted to Google Forms for data collection. All participants were asked to complete a baseline questionnaire scoring Insomnia Severity Index (ISI) [12], Pittsburgh Sleep Quality Index (PSQI) [13], and 5-D Itch Scale (5DIS) [14]; patients with psoriasis were furthermore asked to complete the Dermatology Life Quality Index (DLQI) questionnaire [15]. The same questionnaires were to be completed again after week 4 and week 8.

### Recruitment, Retention, and Costs

The recruitment rate was calculated as the number of participants enrolled in the study on average per day of the active recruitment period. The conversion rate was calculated as the percentage of participants screened who proceeded to be enrolled in the study. The retention rate was calculated as the percentage of participants filling out all the given questionnaires after enrollment.

The budget for advertisements on Google and Facebook was used to calculate the cost per unique view on the website, completed sign-ups, completed baseline questionnaires, and participants completing the entire study.

### Clinical End Points and Statistical Analysis

To assess the correlations between sleep quality, life quality, and itch in the psoriasis group, the total scores from each questionnaire (ie, PSQI, ISI, 5-DIS, and DLQI) were used. When comparing participants with itch, those responding “no itch” were assigned a total score of 5, since this is the equivalent response on the questionnaire. To test for time or group effect for each questionnaire outcome, a linear mixed effects model, presented in an ANOVA table, was applied; the time category was considered as the within-subjects factor, and the psoriasis versus healthy controls groups were treated as the between-subjects factor; the interaction between the group variable and time variable was also assessed to determine if there was a significant change between people living with psoriasis and healthy controls over time. The linear mixed effects models were fitted by a restricted maximum likelihood with the lmer function from the lme4 R package. Visual inspection of quantile-quantile plots and residual plots was performed to reveal any obvious deviations from normality or homoscedasticity. Wilcoxon signed-rank test was conducted to compare average itch scores in good and poor sleepers. The participants were categorized into groups based on their sleep quality: poor sleep (PSQI>5) and good sleep (PSQI≤5) [13]. Statistical analysis was performed using the computing environment R (version 3.6.2; R Core Team).

### Ethics Approval

This study was carried out in accordance with the principles expressed in the Declaration of Helsinki, and the regional ethical committee for the capital region of Denmark was consulted prior to its initiation, which waived the need for ethics approval, as it is a noninterventional questionnaire study (VEK protocol 16025688).

## Results

### Recruitment, Retention, and Costs

Participants were recruited during 2 weeks in September 2016, and data collection was finalized in January 2017. In just 14 days, 12,887 individuals clicked on the ads; 1466 completed the sign-up and screening processes, of which 839 completed the baseline questionnaire and were enrolled in the study. Participants were recruited from 11 different countries on 4 separate continents. The recruitment rate was 59.9 participants per day, and the conversion rate was 57.2%.

Of the 839 participants enrolled, 507 completed the 12-week study: 220 in the psoriasis group and 287 in the control group, with a retention rate of 60.4%.

Study participants were predominantly female and came from multiple different geographical locations (Table 1).

**Figure 1:**
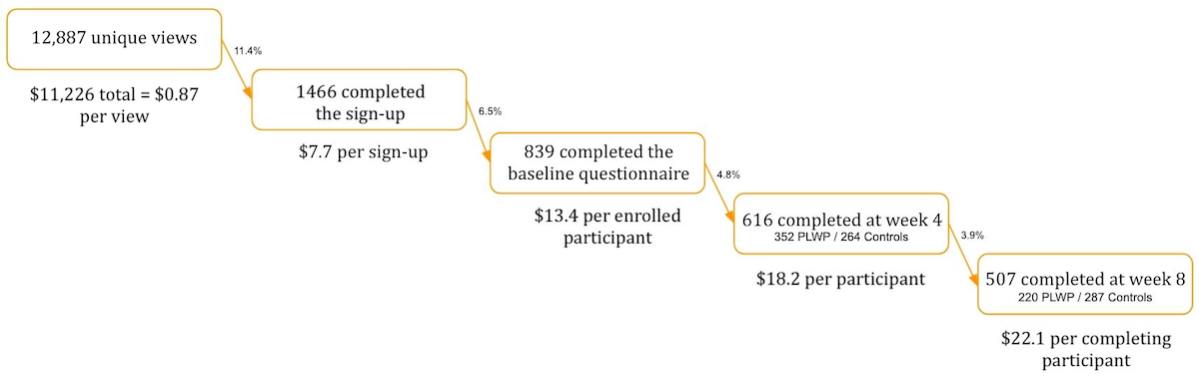
Recruitment, retention, and cost. The currency is in US dollars. PLWP: people living with psoriasis.

The total budget for recruitment was US $11,226 (equivalent to DKK 74,840 on September 1, 2016) [16], translating to US $13.24 per enrolled participant and US $22.1 per participant completing the study (Figure 1).

**Table 1:** Participant characteristics.

Characteristics		Healthy controls (n=287)	Patients with psoriasis (n=220)	*P* value	
Age (years), mean (IQR)		39 (23-55)	49 (38-58)	<.001	
**Gender, n (%)**					.4
	Female	208 (72)	158 (72)		
	Male	76 (26)	62 (28)		
	Other	3 (1)	0 (0)		
**Country, n (%)**					.05
	United Kingdom	119 (41)	91 (41)		
	Canada	93 (32)	80 (36)		
	United States	36 (13)	27 (12)		
	Ireland	36 (13)	14 (6.4)		
	Other^a^	3 (1)	8 (3.6)		
Living with psoriasis (years), mean (IQR)		N/A^b^	18 (6-30)		
**Comorbidities, n (%)**					
	Any	178 (62)	145 (66)	.4	
	None	109 (38)	75 (34)	.4	
	Depression	91 (32)	61 (28)	.3	
	Eczema	20 (7)	18 (8.2)	.6	
**Self-perceived severity of psoriasis, n (%)**					N/A
	Moderate	N/A	126 (57)		
	Mild	N/A	64 (29)		
	Severe	N/A	30 (14)		

^a^Other countries: Denmark, Iran, Italy, Mauritius, New Zealand, and South Africa.

^b^N/A: not applicable.

### Clinical End Points

**Figure 2:**
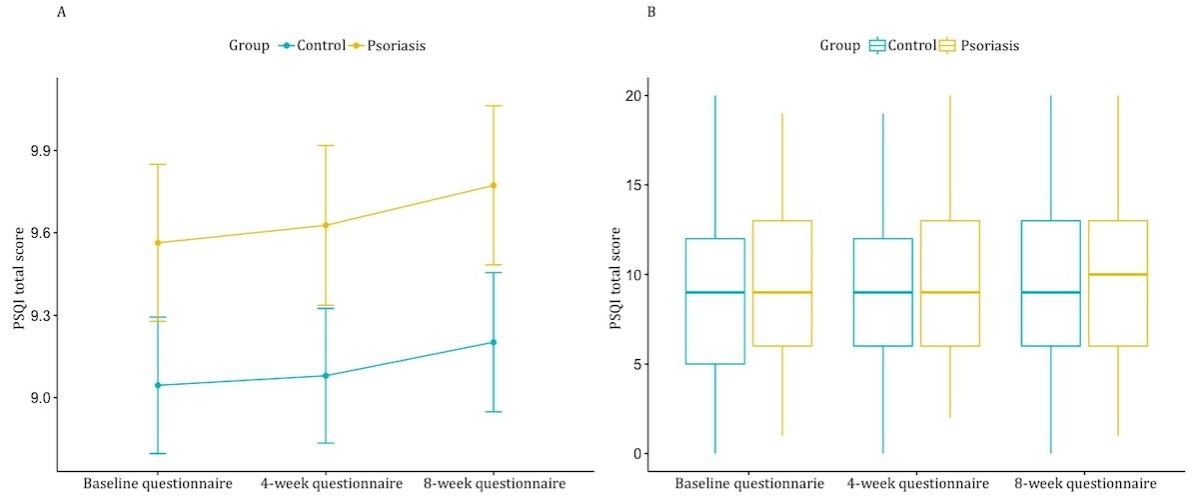
Difference in sleep quality scores between the psoriasis group and the control group over time. (A) Pittsburgh Sleep Quality Index (PSQI) total scores (y-axis) over 3 time points grouped by controls (blue) and patients with psoriasis (yellow). No statistically significant differences were observed. (B) Boxplot of PSQI scores (y-axis) grouped by controls (blue) and patients with psoriasis (yellow). No statistically significant differences were observed.

There were no differences in sleep quality and sleep disturbance between patients with psoriasis and healthy controls over time, assessed with the PSQI questionnaire (Table 2 and Figure 2).

**Figure 3:**
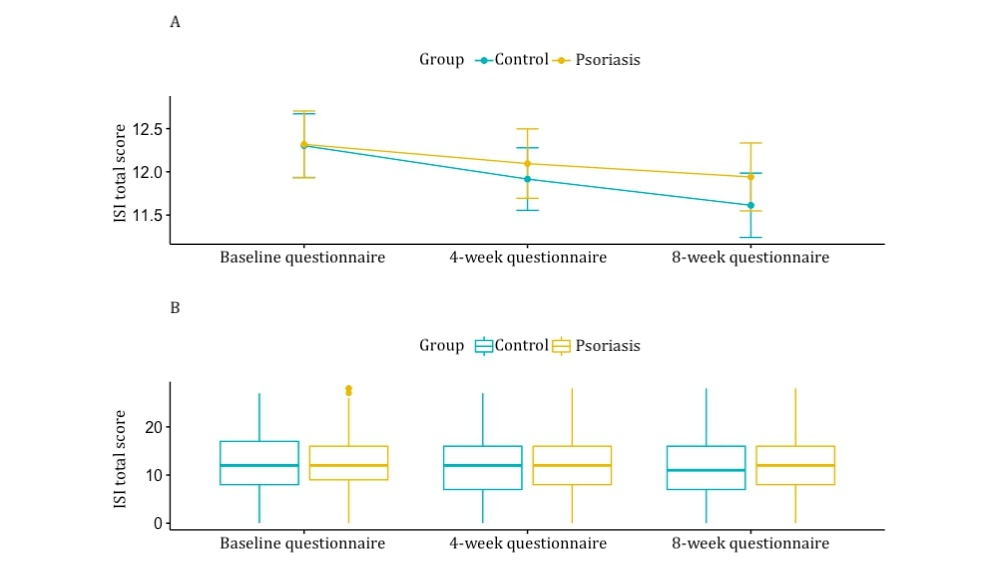
Difference in insomnia scores between the psoriasis group and the control group over time. (A) The mean Insomnia Severity Index (ISI) total scores (y-axis) over time in the control group (blue) and the psoriasis group (yellow). (B) Boxplot of the ISI total scores (y-axis) over time for the control group (blue) and the psoriasis group (yellow).

Moreover, perceived insomnia severity (based on ISI) was not different between groups, but there was a statistically significant decrease in insomnia severity over time within both groups (*P*=.003; Table 2 and Figure 3).

**Figure 4:**
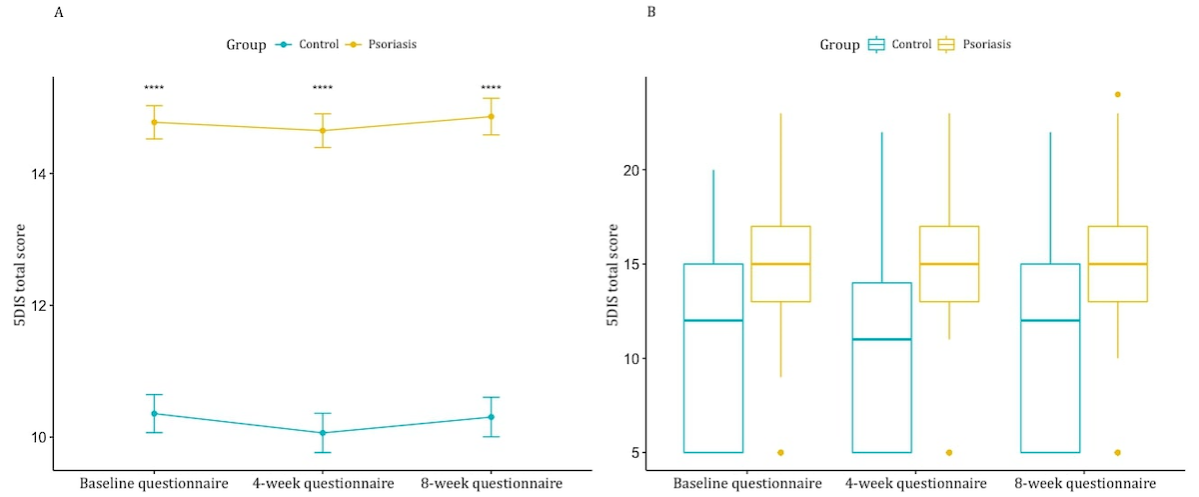
Difference in itch between the psoriasis group and the control group over time. (A) The mean 5D Itch Scale (5DIS) total score (y-axis) over time in the control group (blue) and the psoriasis group (yellow). (B) Boxplot of 5DIS total scores (y-axis) over time (x-axis) for both groups.

Self-assessed itch, reported with the 5-DIS, showed a statistically significant difference between the psoriasis group and the healthy controls (*P*<.001), while there was no statistically significant change over time (Figure 4).

**Figure 5:**
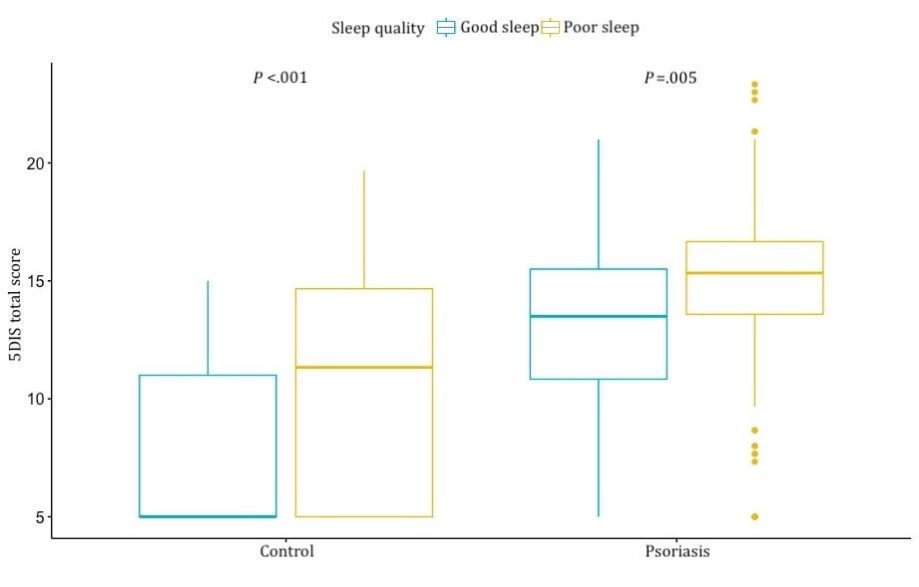
Boxplot of the 5D Itch Scale (5DIS) total score (y-axis) between controls and patients with psoriasis, grouped by the Pittsburgh Sleep Quality Index (PSQI) global score sleep quality. Adhering to the PSQI global score, poor sleep is defined as a score of 5 or more (blue), while good sleep is defined as a score lower than 5 (yellow).

In addition, there was a statistically significant positive correlation between itch and poor sleep in the psoriasis group and the control group (Figure 5), although patients with psoriasis with good sleep generally reported more itchiness, with a median score of 13.5 (IQR 10.8-15.5), compared to the controls with poor sleep, who had a median score of 11.3 (IQR 5.0-14.7).

**Figure 6:**
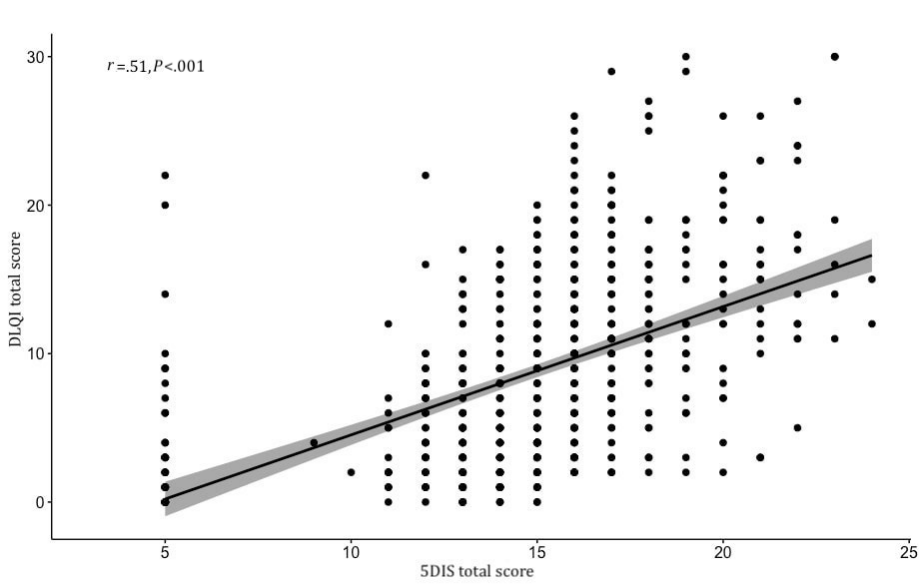
Impaired quality of life as a function of itch. Dermatology Life Quality Index (DLQI) total score is a function of the 5D Itch Scale (5DIS) total score.

There was a moderate correlation between itch and quality of life, as assessed with DLQI (*r*=0.51; Figure 6).

**Table 2:** ANOVA table with *P* values for the fixed effects (ie, time, group, and interaction) for different outcome measures from the questionnaires.

Outcome measure	Time effect		Group effect		Interaction effect	
	*F* value	*P* value	*F* value	*P* value	*F* value	*P* value
PSQI^a^	1.26	.28	2.37	.12	.024	.98
ISI^b^	5.96	.003	.12	.73	.51	.60
5DIS^c^	1.46	.23	148.75	<.001	.18	.84

^a^PSQI: Pittsburgh Sleep Quality Index.

^b^ISI: Insomnia Severity Index.

^c^5DIS: 5D Itch Scale.

## Discussion

### Principal Findings

In just 2 weeks, we recruited 1466 participants from several countries into an 8-week observational study of psoriasis. The conversion rate was 58%, and the retention rate was almost 60%, meeting the goal of 500 participants with a total of 507 participants completing the entire study. It is not uncommon to experience dropout rates as high as 70% in longitudinal studies [17], and considering the lack of in-person visits and the extensive study design with time-consuming questionnaires for study participants over the course of 8 weeks, our figures were surprisingly high. We managed to recruit participants from 11 different countries on 4 separate continents, offering a huge potential for representative and generalizable data.

In a recent systematic review and meta-analysis assessing cost-effectiveness of web-based and in-person recruitment strategies in 23 studies, the median cost per participant enrolled via the internet was US $72, while the median cost per enrollee for in-person recruitment was US $199 [10]. Although the study designs and the eligibility criteria for study participants varied tremendously, the cost per enrolled participant in this study was US $13, making it an extremely cost-effective approach. Pricing for Google Ads and Facebook Ads also varies over time, making it difficult to compare cost-effectiveness.

Although our clinical end points served as retention catalysts in this feasibility study, the questionnaires also offered information on sleep quality or insomnia, itch, and for patients with psoriasis, quality of life. We were able to show statistically significant differences in itch between patients with psoriasis and healthy controls, a common characteristic of psoriasis [18]. We also found that sleep quality as assessed by the total score in the ISI questionnaire improved from baseline to week 4 and from week 4 to week 8. In previous studies, the ISI was only moderately correlated with the PSQI [19], which could explain the lack of similar changes over time in the PSQI total score. Lower sleep quality is associated with other comorbidities, such as depression [20]; however, the 2 groups did not show a statistically significant difference in this parameter.

Even though this study was conducted in 2016, the general methods for web-based patient recruitments have not changed drastically, Facebook still being the most commonly used social media recruitment platform [7]. TikTok and other more recent platforms tend to cater to younger audiences not usually enrolled in clinical studies [21,22].

### Study Limitations

There are several limitations in this study. With the potential of heterogenous and generalizable data, collected using web-based recruitment, it is important to mention the potential opposite effect. A recent study from 2022 by Li et al [23] investigating recruitment and retention within different incentive structures in the United States found certain characteristics related to higher retention rates. Overall, older participants showed higher retention rates. In a subgroup, where participants were recruited from advertisements in social media and web-based newspapers, ethnicity (non-Hispanic White), education level (college or higher) and income level (<US $49,999) showed longer retention. These findings suggest the necessity of finding other methods in retaining a more diverse study population.

Another limitation of our study is that we did not assess how demographic data of study participants pertained to study outcomes, such as recruitment and retention. One notable difference from Li et al [23] is that we did not offer monetary incentives. This may likely affect the demographic characteristics of the study population, especially regarding income levels. Future studies should evaluate whether their recruitment outcomes are related to participant demographics.

Given the self-reported design of this study, one limitation relates to our secondary, clinical outcomes. There is a possibility of recruiting patients without a clinically validated psoriasis diagnosis. There is also the possibility of a selection bias toward patients having more severe psoriasis than is representable in the standard population. Although patients were asked whether they were clinically diagnosed by a certified physician, the self-perceived severity of their diagnosis, and the treatment options they used, there was no way to verify this information.

One major limitation to web-based recruitment is selection bias. In our study, focusing on psoriasis and sleep quality, the healthy controls most likely had an interest in sleep quality, and that presented a possible bias. Another limitation was thought to be related to age, with the hypothesis that older people, being less digitally savvy, would not sign up because of the fully remote digital design of the study. However, the median age in the psoriasis group was 49 years, while it was 39 years in the healthy control group; given the bimodal peaks of psoriasis onset being 20-30 and 50-60 years of age, the age group in this study was representative.

The observational design of this study also offers limitations when compared to decentralized clinical trials with interventional elements. Comparing the number of sign-ups to the completion of the questionnaires, it is possible that the easy sign-up process might lead to higher dropout rates later in the study.

### Conclusions

In conclusion, we managed to enroll and retain more than 500 study participants not restricted by geography for a duration of 8 weeks in this feasibility study. This represents a promising strategy for recruitment of patients with rare diseases and to furthering better representation of study participants. Future studies should investigate the feasibility of web-based recruitment in clinical trials without geographical restrictions and explore the characteristics of study populations recruited through this method.

## Appendix

Multimedia Appendix 1 Google AdWords.

Multimedia Appendix 2 Sign-up questionnaire.

## Abbreviations

5DIS: 5D Itch Scale
DLQI: Dermatology Life Quality Index
ISI: Insomnia Severity Index
PSQI: Pittsburgh Sleep Quality Index
