# Supplementary material for: Rapid Web-Based Recruitment of Patients With Psoriasis: Multinational Cohort Study
Source: JMIR Dermatol. 2023 Jun 20;6:e44405. doi: 10.2196/44405 (PMC10335121; doi:10.2196/44405)
Supplement: Multimedia Appendix 1 [file derma_v6i1e44405_app1.docx]

Appendix 1: Google Adwords

The Google Adword campaign used modified broadmatch as a targeting option, enabling participants to search for “anything + psoriasis” to trigger an advert. The adverts were displayed as top line results when using the search engine (see Table 3 for examples).

The Google Display Ads (ads on webpages) were targeted towards two primary groups; patients with psoriasis and people without psoriasis (control group). Websites with content concerning psoriasis and dermatology were used to recruit patients with psoriasis. Campaign adverts in the Facebook newsfeed were targeted towards women and men, over 18 years of age, with an interest in psoriasis. For the control group, women and men, over 18 years of age, were targeted.

Table 3. Adcopies

| Adcopy for Control group: | *Headline: Join Our Sleep Study*  Description: We’re conducting a study on how sleep affects people. Read about it and apply to our new study right here: <link> |
| --- | --- |
| Adcopy for Psoriasis group: | *Headline: Join Our Study on Psoriasis and Sleep*  Description: We’re conducting a study on the relations between sleep and psoriasis. Follow this link to learn more and apply for our 8-week study: <link> |
