# Supplementary material for: Rapid Web-Based Recruitment of Patients With Psoriasis: Multinational Cohort Study
Source: JMIR Dermatol. 2023 Jun 20;6:e44405. doi: 10.2196/44405 (PMC10335121; doi:10.2196/44405)
Supplement: Multimedia Appendix 2 [file derma_v6i1e44405_app2.docx]

Appendix 2: Signup Questionnaire

1. What is your first name?
2. What is your gender?
   1. Female/Male/Other
3. What is your age?
   1. 18-80
4. How tall are you? (CM/Inches)
5. How much do you weigh? (KG/LBS)
6. Do you read and write English fluently?
   1. Yes/No
7. **Have you been diagnosed with psoriasis by a certified physician**
   1. Yes/No
      1. *Skip psoriasis questions if “no”.
8. How many years have you had psoriasis?
   1. 1-80
9. What kind of psoriasis do you have?
   - 1. Checkbox
        1. Plaque Psoriasis
        2. Inverse Psoriasis (In skin folds)
        3. Nail Psoriasis
        4. Psoriatic Arthritis
        5. Guttate Psoriasis
        6. Pustular Psoriasis
        7. Erythrodermic Psoriasis
        8. I don't know
10. How would you rate the severity of your psoriasis?
    - - 1. Mild
        2. Moderate
        3. Severe
11. How big a percentage of your skin would you estimate is affected by psoriasis?
    1. The palm of your hand can serve as a reference point. Your palm is roughly equivalent to 1% of your total skin surface area.
       1. For example:
          1. 1-3%
          2. 4-10%
          3. 11-20%
          4. 21-30%
          5. 31-40%
          6. 41-50%
          7. 51-60%
          8. 61-70%
          9. 71-80%
          10. 81-90%
          11. 91-100%
12. Which of the following body areas are inflicted by your psoriasis?
    - - 1. Face
        2. Neck
        3. Hands
        4. Knees
        5. Lower legs
        6. Feet
        7. Arms
        8. Shoulders
        9. Scalp
        10. Chest
        11. Stomach
        12. Back
        13. Thighs
        14. Genital area
        15. Anal area
        16. Other...
13. Have you been diagnosed with any conditions?
    - 1. We need to see if people with different comorbidities have different problems with sleep.
         1. Psoriasis
         2. Eczema
         3. Diabetes
         4. Cardiovascular Disease
         5. Crohn's Disease
         6. Depression
         7. Metabolic Syndrome
         8. Obesity
         9. Osteoporosis
         10. Uveitis
         11. Liver Disease
         12. Arthritis
         13. Sleep related diseases (like apnoea and insomnia)
         14. Other…
14. Does your skin itch?
15. Do you use any medications and if so, how often do you use it?
    - 1. Eg. Clobetasol, once daily.
         1. Long answer text
16. Are you willing to and interested in answering four questions every week regarding your sleep quality?
    - 1. It will be simple questions where you answer with a number from 0-9, e.g., how many hours you on average did sleep.
    1. Yes/No
17. Are you willing to and interested in answering 3 larger questionnaires, once a month, for 2 months regarding your sleep patterns, your health and your quality of life?
    1. Yes/No
18. Do you have, and use, a personal cell phone where you can receive and send texts?
    1. Yes/No
19. What is your cell phone number?
    - 1. Number field
20. What is your e-mail Address?
    1. Please use a private email address that you check regularly as we will run most of the communication through this.
       1. Repeat your email address.
21. What is your full address and country
    1. Would you be willing to share your geographical location for analysis purposes, so that we can see if temperature, light hours of the day and humidity is influencing your quality of sleep?

Contract Consent
